# Child Intelligence and Reductions in Water Arsenic and Manganese: A Two-Year Follow-up Study in Bangladesh

**DOI:** 10.1289/ehp.1509974

**Published:** 2015-12-29

**Authors:** Gail A. Wasserman, Xinhua Liu, Faruque Parvez, Pam Factor-Litvak, Jennie Kline, Abu B. Siddique, Hasan Shahriar, Mohammed Nasir Uddin, Alexander van Geen, Jacob L. Mey, Olgica Balac, Joseph H. Graziano

**Affiliations:** 1 Department of Psychiatry, College of Physicians and Surgeons, Columbia University, New York, New York, USA; 2 New York State Psychiatric Institute, New York, New York, USA; 3 Department of Biostatistics,; 4 Department of Environmental Health Sciences, and; 5 Department of Epidemiology, Columbia University Mailman School of Public Health, New York, New York, USA; 6 Columbia University Arsenic Project Office, Mohakhali, Dhaka, Bangladesh; 7 University of Chicago Research Office, Mohakhali, Dhaka, Bangladesh; 8 Lamont-Doherty Earth Observatory, Columbia University, New York, New York, USA

## Abstract

**Background:**

Arsenic (As) exposure from drinking water is associated with modest intellectual deficits in childhood. It is not known whether reducing exposure is associated with improved intelligence.

**Objective:**

We aimed to determine whether reducing As exposure is associated with improved child intellectual outcomes.

**Methods:**

Three hundred three 10-year-old children drinking from household wells with a wide range of As concentrations were enrolled at baseline. In the subsequent year, deep community wells, low in As, were installed in villages of children whose original wells had high water As (WAs ≥ 50 μg/L). For 296 children, intelligence was assessed by WISC-IV (Wechsler Intelligence Scale for Children, 4th ed.), with a version modified for the study population, at baseline and approximately 2 years later; analyses considered standardized scores for both Full Scale IQ and Verbal Comprehension, Perceptual Reasoning, Working Memory, and Processing Speed Indices. Creatinine-adjusted urinary arsenic (UAs/Cr), blood As (BAs), and blood manganese (BMn) were assessed at both times.

**Results:**

UAs/Cr concentrations declined significantly by follow-up for both the high (≥ 50 μg/L) and low (< 50 μg/L) WAs subgroups. At baseline, adjusting for maternal age and intelligence, plasma ferritin, head circumference, home environment quality, school grade, and BMn, UAs/Cr was significantly negatively associated with Full Scale IQ, and with all Index scores (except Processing Speed). After adjustment for baseline Working Memory scores and school grade, each 100-μg/g reduction in UAs/Cr from baseline to follow-up was associated with a 0.91 point increase in Working Memory (95% CI: 0.14, 1.67). The change in UAs/Cr across follow-up was not significantly associated with changes in Full Scale IQ or Index scores.

**Conclusions:**

Installation of deep, low-As community wells lowered UAs, BAs, and BMn. A greater decrease in UAs/Cr was associated with greater improvements in Working Memory scores, but not with a greater improvement in Full Scale IQ.

**Citation:**

Wasserman GA, Liu X, Parvez F, Factor-Litvak P, Kline J, Siddique AB, Shahriar H, Uddin MN, van Geen A, Mey JL, Balac O, Graziano JH. 2016. Child intelligence and reductions in water arsenic and manganese: a two-year follow-up study in Bangladesh. Environ Health Perspect 124:1114–1120; http://dx.doi.org/10.1289/ehp.1509974

## Introduction

Exposure to naturally occurring arsenic (As) in drinking water is a global environmental health problem. The World Health Organization (WHO) estimates that > 200 million people worldwide are chronically exposed to levels above the WHO and U.S. Environmental Protection Agency safety standard of 10 μg/L (WHO 2008). Arsenic is a carcinogen associated with lung, bladder, skin, and possibly other cancers. It has also been associated with cardiovascular disease, skin lesions, nonmalignant respiratory disease, and deficits in child development (reviewed by Naujokas et al. 2013).

Over the past decade, we and others have become increasingly concerned that exposures to As and manganese (Mn) in drinking water in household wells are associated with decreases in children’s intellectual functioning. For example, in our earlier work in Bangladesh with 6- (Wasserman et al. 2007) and 10-year olds (Wasserman et al. 2004), after adjustment for social factors related to intellectual function, water arsenic concentration (WAs) was inversely associated with WPPSI-III (Wechsler Preschool and Primary Scale of Intelligence, 3rd ed.) and WISC-III (Wechsler Intelligence Scale for Children, 3rd ed.) scores, using instruments that were modified for the study population. In 2006, we also demonstrated that 10-year-old children with low WAs but elevated water manganese concentrations (WMn) had lower intelligence scores (Wasserman et al. 2006). In a neighboring region in West Bengal (von Ehrenstein et al. 2007) and in two studies of children residing near smelter complexes in Mexico (Calderón et al. 2001; Rosado et al. 2007), urinary arsenic (UAs) concentration was negatively related to some subtests of the WISC. In a small study relating hair levels of various metals to U.S. children’s intelligence (Wright et al. 2006), As concentration in hair was associated with poorer verbal learning and memory scores. In a recent large study in Bangladesh (Hamadani et al. 2011), WAs exposure at age 5 years was associated with lower scores in both Full Scale IQ (FSIQ) and Verbal intelligence (WPPSI-III; Wechsler 2002).

Global health researchers and practitioners alike have developed and investigated a variety of strategies aimed at mitigating As exposure, such as community-based programs focused on well-switching (Ahmed et al. 2006; Chen et al. 2007; George et al. 2012), school-based educational interventions (Khan et al. 2015), and programs encouraging families’ use of filters (Peters et al. 2015). Those programs have shown modest success, in terms of both reported water usage behavior, and changes in the biomarkers that reflect changes in the consumption of As via water.

To evaluate the degree to which switching to wells with lower WAs would be associated with improvements in children’s intellectual functioning, we recruited 303 8- to 11-year-old children between January and December 2008. The study involves children of adults who are participants of the Health Effects of Arsenic Longitudinal Study (HEALS; Ahsan et al. 2006). Children were selected to fall into four strata defined by high/low As (WAs > 10 μg/L vs. WAs ≤ 10 μg/L) and high/low Mn (WMn > 500 μg/L vs. WMn ≤ 500 μg/L) concentrations of their household wells. Earlier, we reported on assessments of child intelligence among these children at baseline (Wasserman et al. 2011). Following those assessments, 58 deep community wells were installed for children for whom WAs in home wells exceeded 50 μg/L (i.e., the Bangladesh standard); all but two of the newly installed community wells met the WHO guideline of 10 μg/L, and all met the Bangladesh national standard of 50 μg/L. Although our primary focus was on WAs, these installations also reduced WMn exposure, which is correlated with WAs in wells in our study region (Wasserman et al. 2004). Further, the Bangladesh government has installed and continues to install deep community wells throughout the country, including over the duration of this particular study in Araihazar (van Geen et al. 2016). Here, we report on follow-up assessments of 296 of those children.

## Materials and Methods

### Study Timeline

Initial baseline recruitment and evaluation took place between January 2008 and December 2008. Deep well installation occurred between January 2009 and December 2009. The present follow-up evaluation of study participants took place between January 2010 and December 2010.

### Participants

As noted earlier (Wasserman et al. 2011), following a survey of the well characteristics of HEALS cohort villages within commuting distance of our field clinic, we designated household wells into one of four groups: high As/high Mn (WAs > 10 μg/L and Mn > 500 μg/L), high As/low Mn, low As/high Mn, and low As/low Mn. At baseline, we identified all children estimated to be between 8 and 11 years old, and recruited at random until approximately 75 children were included in each of the four well categories. Inclusion criteria restricted enrollment to children 8–11 years of age who attended school in an age-appropriate grade, who had no known physical disability or chronic illness, who were not twins and who did not share a home well with other child participants. Children were equally divided by gender.

The baseline sample consisted of 303 participants (299 of whom agreed to a blood draw for BAs and BMn). All 303 were relocated for the follow-up, and 296 were reassessed in the field clinic, 278 of whom agreed to a blood draw. Seven children were not seen at follow-up because they had moved away from the region.

### Procedure

This study was approved by the institutional review boards of Columbia University Medical Center and the Bangladesh Medical Research Council. During baseline home visits, once informed parental consent and child assent were obtained, the field team collected sociodemographic information, and appointments were made for mother and child to visit the field clinic. At baseline and follow-up field clinic visits, a physical examination and a neurodevelopmental assessment were done; urine and blood samples were collected. Children received a small age-appropriate gift in appreciation for participation.

Over the 12 months after the baseline assessment, in a collaboration with Water Aid, Bangladesh, we installed 58 community wells averaging 225 m in depth supplying groundwater with average As and Mn content of 2.9 μg/L and 0.45 mg/L, respectively, in the villages of children in the two high As quadrants. A blanket survey of nearly 50,000 wells in this region in 2012–2013 (van Geen et al. 2014) revealed that the Bangladesh government had installed ~ 700 deep wells in the HEALS study area, so that exposure reduction likely occurred for families beyond the ones for whom we intervened by providing new wells. Families were revisited at home to schedule the follow-up visit to the field clinic. On average, the installation of a new well took place 12 months after the baseline assessment, and the follow-up assessment 2.79 years after baseline.

### Measures

Information about mother’s well use was collected when these families were recruited into the HEALS cohort expansion (2006–2008). Recruitment for the baseline assessments of the present study (Wasserman et al. 2011) was limited to families who had not switched wells since they were recruited into HEALS. At the time of our baseline assessments, almost all (*n* = 297, 97.7%) mothers of participants reported drinking exclusively from the index well, and 78% had drunk from that well for ≥ 5 years. We were unable to systematically obtain information about well use at follow-up and therefore relied on biomarkers of exposure. Field sample collection and laboratory analysis procedures are described elsewhere in detail (Cheng et al. 2004; van Geen et al. 2003, 2005).

***Urinary measurements.*** Total UAs concentrations were assayed by graphite furnace atomic absorption spectrophotometry (GFAA), using a Perkin-Elmer Analyst 600 system as described (Nixon et al. 1991). UAs levels were also adjusted for urinary creatinine (UCr) concentrations (by dividing UAs by creatinine levels), which were analyzed by a colorimetric method based on Jaffe’s reaction (Heinegård and Tiderström 1973).

***ICP-MS blood measurements.*** Venous whole blood samples were analyzed for BAs and BMn concentrations using a Perkin-Elmer Elan DRC II ICP-MS (inductively coupled plasma mass spectrometer) equipped with an AS 93+ auto sampler. ICP-MS-DRC methods for metals in whole blood were developed according to published procedures (Pruszkowski et al. 1998; Stroh 1988). The intraprecision coefficients of variation for blood manganese (BMn) and blood arsenic (BAs) were 3.1% and 3.3%, respectively, and the interprecision coefficients were 6.3% and 7.7%, respectively. Venous blood samples were used for measuring hemoglobin (Hgb) and serum ferritin (SF) (Miles et al. 1974).

***Child intelligence.*** The Wechsler Intelligence Scale for Children, Fourth Edition (WISC-IV; Wechsler 2003) is an individually administered assessment of intellectual function, suitable for children 6–16 years old. This revised version of the WISC-III (Wechsler 1991) has excellent psychometrics (e.g., mean standard errors of measurement for 15-year-olds for the subtests used were 1.13), and provides measures of general intellectual ability (Full Scale IQ) and specific cognitive domains (Verbal Comprehension, Perceptual Reasoning, Working Memory, and Processing Speed Indices). We administered a battery of the following subtests (listed with their respective composites): Similarities, Comprehension, and Information (Verbal Comprehension); Block Design, Matrix Reasoning, and Picture Completion (Perceptual Reasoning); Digit Span and Letter-Number Sequencing (Working Memory); Coding and Symbol Search (Processing Speed). As described in our earlier work, we modified certain test items that were thought to be culturally inappropriate (e.g., we did not administer Comprehension items referring to seatbelts or libraries because villagers rarely ride in automobiles, and there are no local public libraries) (Wasserman et al. 2011).

We measured maternal intelligence at baseline on the Wechsler Abbreviated Scale of Intelligence (WASI; Wechsler 1999), a short and reliable measure of intelligence, across the age span. It consists of two Performance subtests (Block Design and Matrix Reasoning) and two Verbal (Vocabulary and Similarities) subtests. Our battery included Vocabulary and Matrix Reasoning subtests. Cultural considerations prompted slight changes to the Vocabulary subtest: For example, we eliminated the definition of a “cart,” a vehicle not used in this setting.

We assessed sociodemographic characteristics during a structured interview with a parent during the baseline home visit, including maternal and paternal age, ethnicity, education, and occupation. At both baseline and follow-up visits, mothers were asked for how many months the child had attended school. Child’s height, weight, and head circumference were measured at the clinic visit after the WISC-IV.

***Home environment.*** During the baseline home visit, we obtained information about six items that described enriching materials and opportunities available to the child at home (see Wasserman et al. 2011), as an analogue for a measure of the home environment found to contribute to child intelligence (HOME; Caldwell and Bradley 1984, 2001). The items included presence of a clock in the home, displayed artwork or a current wall calendar, availability in the immediate household family of a watch, presence of any age-appropriate toy, and whether the child had made a trip away from the region in the preceding 6 months.

### Translation and Training

All tests and interviews were translated (and back-translated) between Bangla (Bengali) and English with the incorporation of culturally appropriate adaptations (Wasserman et al. 2011). Three testers were trained (by G.W.) and continued with supervised practice sessions for 2 weeks. All written test responses were rechecked for valid ranges and missing data when sent to Columbia University for entry and analysis.

### Data Analysis

To examine the impact of well installation on As exposure, we first examined associations between changes between baseline and follow-up levels of UAs/Cr and BAs and information on well accessibility as reflected in *a*) household distance to the 58 deep, low-As wells expressly installed for this study by our collaborator (Water Aid, Bangladesh) and *b*) the distance (from the child’s home) to any deep, low-As well, regardless of which organization had installed the well (based on our 2012–2013 well installation survey) (van Geen et al. 2014). We did this because we previously observed that distance between a safe well and the home is indeed a predictor of UAs (Chen et al. 2007; George et al. 2012).

Next, we generated standardized Index and IQ scores as outcomes, rather than the raw scores (weighted sums of items successfully passed) relied on earlier in studies with this population (Wasserman et al. 2004, 2006, 2007, 2011). The use of standardized scores allowed us to estimate changes in children’s scores over time that are independent of changes associated with increasing age (because standardized scores take age into account) and to facilitate comparisons with other study populations. Because our earlier work (Wasserman et al. 2011) considered raw WISC-IV scores, rather than the standardized scores relied on here, we first sought to establish that associations between baseline features and standardized measures of IQ and Index scores were similar to those previously found for raw scores. Following the same steps as in developing that baseline model (Wasserman et al. 2011), we used multiple regression to relate WISC-IV scores to BAs and BMn, net of contributions of maternal age and intelligence, plasma ferritin, head circumference, quality of the HOME environment, and school grade.

We then used regression models to estimate associations between natural log–transformed UAs/Cr or natural log–transformed BAs at baseline and standardized WISC-IV scores at baseline (FSIQ and Verbal Comprehension, Working Memory, Perceptual Reasoning, and Processing Speed subscales), adjusting for baseline factors that were statistically significant predictors of either outcomes at baseline (*p* < 0.05), specifically, maternal intelligence, maternal age, HOME score, child’s head circumference, school grade at baseline, plasma ferritin [categorized as high (> 32.5), low (≤ 32.5), or not available, with low used as the reference group], and natural log–transformed BMn. All covariates other than plasma ferritin were modeled as continuous variables.

We also used linear regression models to examine the associations between the change in UAs/Cr (or BAs) from baseline to follow-up, and the change in standardized WISC scores between baseline and follow-up. Models for changes from baseline to follow-up were adjusted for the corresponding baseline outcome score as an indicator of baseline intelligence (e.g., models of the change in FSIQ were adjusted for baseline FSIQ, and models of the change in Verbal Comprehension Index were adjusted for baseline Verbal Comprehension Index) and for school grade at baseline (the only other significant predictor of the change in outcomes).

Initial contributory analyses also considered the role of changes in BMn, and the interactions of the changes in As by changes in BMn, with each term added in turn to the prior models as described above for UAs/Cr and BAs. Neither changes in BMn nor the BAs by BMn interaction measure contributed significantly to changes in IQ test scores.

## Results

Of the 303 children evaluated at baseline, all of whom were attending school at that time, 296 were seen at follow-up, nearly 3 years later. The mean (± SD) ages at baseline and follow-up were 9.64 ± 0.77 years and 12.43 ± 0.78 years, respectively. In each case the participants were 49–50% male. At baseline, mothers’ mean age was 35.30 ± 6.02 years of age and mothers had 3.74 ± 3.69 years of education and a mean WASI score of 31.00 ± 10.60.

### Change in Exposure Biomarkers

Urinary indicators of As exposure (UAs and UAs/Cr) were lower at follow-up for the entire sample (within-person mean decreases of 19.81 μg/L and 118.36 μg/g, *n* = 292, respectively, both *p* < 0.0001), with greater decreases in the high-WAs group (Table 1). Similarly, the switch to lower-As wells resulted in significantly decreased levels of both BAs and BMn for the entire sample (mean decreases of 0.44 μg/L and 1.53 μg/L, *p* < 0.05 and *p* < 0.0001, respectively) with the decreases in both BAs and BMn larger in the high-WAs group (*p* < 0.0001).

**Table 1:** Exposures and outcomes at baseline and follow-up, and changes over time, according to baseline water As (mean ± SD).

Exposure	Baseline WAs > 10 μg/L			Baseline WAs ≤ 10 μg/L			Total		
	Baseline *n* = 152	Follow-up *n* = 150	Change *n* = 150	Baseline *n* = 151	Follow-up *n* = 146	Change *n* = 146	Baseline *n* = 303	Follow-up *n* = 296	Change *n* = 296
Water As (μg/L)	83.5 ± 87.1	NA		2.9 ± 2.6	NA		43.3 ± 73.7	NA
Water Mn (μg/L)	798.9 ± 780.8	NA		647.6 ± 669.1	NA		723.5 ± 730.0	NA
Urinary As (μg/L)^*a*^	106.6 ± 86.0	67.7 ± 58.5	–39.68 ± 95.31	49.4 ± 37.3	49.9 ± 41.3	0.62 ± 50.71	78.1 ± 72.2	58.9 ± 51.5	–19.81 ± 79.12
Urine As (μg/g creatinine)^*a*^	331.3 ± 210.2	158.4 ± 102.5	–176.08 ± 190.48	161.2 ± 95.0	102.4 ± 74.7	–59.03 ± 103.68	246.5 ± 183.9	130.8 ± 94.0	–118.36 ± 164.46
Blood As (μg/L)^*b*^	6.3 ± 3.6	4.9 ± 2.7	–1.44 ± 3.77	3.3 ± 1.7	3.9 ± 2.7	0.61 ± 2.48	4.8 ± 3.2	4.4 ± 2.7	–0.44 ± 3.36
Blood Mn (μg/L)^*b*^	14.5 ± 3.4	13.0 ± 3.2	–1.53 ± 1.81	15.0 ± 4.0	13.4 ± 4.1	–1.54 ± 2.25	14.8 ± 3.7	13.2 ± 3.7	–1.53 ± 2.03
WISC-IV IQ
Verbal Comprehension	64.9 ± 10.9	63.0 ± 10.3	–1.24 ± 8.94	64.85 ± 9.9	62.13 ± 8.6	–2.55 ± 7.20	64.9 ± 10.4	62.59 ± 9.5	–1.89 ± 8.14
Perceptual Reasoning	65.1 ± 10.6	65.0 ± 10.0	0.29 ± 8.62	64.6 ± 9.9	64.3 ± 8.5	–0.34 ± 8.36	64.9 ± 10.2	64.7 ± 9.3	–0.02 ± 8.48
Working Memory	76.2 ± 13.9	77.3 ± 16.2	1.59 ± 11.86	76.2 ± 13.3	76.0 ± 14.5	–0.14 ± 10.72	76.2 ± 13.6	76.7 ± 15.4	0.73 ± 11.33
Processing Speed	71.1 ± 11.5	72.7 ± 9.8	1.81 ± 7.42	71.9 ± 10.3	73.3 ± 10.0	1.49 ± 8.86	71.5 ± 10.9	73.0 ± 9.9	1.66 ± 8.15
Full Scale	61.6 ± 11.1	61.6 ± 10.5	0.59 ± 6.23	61.6 ± 10.2	61.0 ± 9.5	–0.64 ± 6.23	61.6 ± 10.6	61.3 ± 10.0	–0.02 ± 6.25
Abbreviations: NA, not applicable; WAs, water arsenic. ^***a***^Total *n* = 292: *n* = 148 with high WAs and *n* = 144 with low WAs. ^***b***^Total *n* = 276: *n* = 141 with high WAs and *n* = 135 with low WAs.									

As noted earlier, we examined the relationship between changes in UAs/Cr and the distance (from the child’s home) to any deep, low-arsenic well. For the entire sample (*n* = 292) and for those with high WAs (> 10 μg/L, *n* = 148) at baseline, there were significant negative correlations between the distance from the home to the nearest deep well and the change in UAs/Cr from baseline to follow-up: *r* = –0.26 (*p* < 0.0001) and *r* = –0.25 (*p* = 0.0023), respectively, for the total population (*n* = 276); and *r* = –0.23 (*p* < 0.01) and *r* = –0.27 (*p* = 0.002), respectively, for those with high WAs at baseline (*n* = 141). Distances to low-As wells were not significantly correlated with changes in BMn, however (|*r*| < 0.10, *p* > 0.25).

### Concurrent Associations of Biomarkers with Baseline IQ

Before adjustment for other contributors, baseline BAs was significantly negatively related to Full Scale IQ and to Index scores, whereas baseline BMn was significantly associated with Full Scale, Working Memory, and Perceptual Reasoning scores (Table 2). Together these explained 2.30–6.64% of the variance in IQ and Index scores. With covariate adjustment, associations with BAs persisted for all scores (except Processing Speed), whereas significant association with BMn persisted only for Working Memory; estimated regression coefficients for both indicators were substantially reduced (by about half) with the inclusion of other terms in predictive models.

**Table 2:** Baseline associations (coefficient and 95% CI) between standardized WISC-IV scores and log_e_-transformed blood As and blood Mn (μg/L, *n* = 299).

Model	Full Scale	Verbal Comprehension	Working Memory	Perceptual Reasoning	Processing Speed
Crude^*a*^
BAs	–3.22 (–5.13, –1.31) *p* = 0.001	–2.88 (–4.78, –0.97) *p* = 0.003	–3.63 (–6.11, –1.15) *p* = 0.004	–2.24 (–4.11, –0.36) *p* = 0.02	–2.24 (–4.28, –0.20) *p* = 0.032
BMn	–6.59 (–11.43, –1.74) *p* = 0.008	–4.76 (–9.60, 0.07) *p* = 0.054	–9.00 (–15.3, –2.69) *p* = 0.005	–6.10 (–10.85, –1.35) *p* = 0.012	–3.19 (–8.37, 1.99) *p* = 0.23
*R*^2^	6.64%	4.65%	5.95%	4.45%	2.30%
Adjusted^*b*^
BAs	–2.27 (–3.91, –0.63) *p* = 0.007	–2.10 (–3.83, –0.37) *p* = 0.018	–2.75 (–4.96, –0.55) *p* = 0.014	–1.60 (–3.37, 0.17) *p* = 0.076	–1.25 (–3.07, 0.56) *p* = 0.17
BMn	–3.27 (–7.51, 0.97) *p* = 0.13	–1.78 (–6.24, 2.68) *p* = 0.43	–6.27 (–11.94, –0.59) *p* = 0.03	–3.55 (–8.11, 1.02) *p* = 0.13	–0.18 (–4.86, 4.50) *p* = 0.94
*R*^2^	35.04%	26.14%	30.54%	19.73%	27.45%
^***a***^Model includes log_e_-BAs and log_e_-BMn only. ^***b***^Additionally adjusted for maternal IQ score and maternal age; HOME score; child’s school grade, head circumference, and plasma ferritin (categorized as low, high, or not available).					

When we next examined the contribution of UAs/Cr to intelligence and Index scores (using this same model), similar patterns were observed in both adjusted and unadjusted analyses (Table 3). Adjusting only for BMn, UAs/Cr was negatively (and significantly) associated with Full Scale IQ, and with all Index scores, except for Perceptual Reasoning. In adjusted analyses, UAs/Cr remained a significant negative contributor to Full Scale IQ, as well as to Working Memory and Verbal Comprehension Indices. For a difference in BAs between 3.31 (the mean BAs in the low-WAs group at baseline) and 6.31 (the baseline mean in the high-WAs group), Full Scale IQ, Verbal Comprehension and Working Memory Indices were lower by 1.47, 1.35 and 1.78 points, respectively. For example, at baseline, Full Scale IQ was 1.47 points lower in the high-WAs group than it was in the low-As group [–2.27 log_e_ (6.31/3.31) = –1.47].

**Table 3:** Baseline associations (coefficient and 95% CI) between standardized WISC-IV scores and log_e_-transformed urinary As/Cr and blood Mn (μg/L, *n* = 299).

Model	Full Scale	Verbal Comprehension	Working Memory	Perceptual Reasoning	Processing Speed
Crude^*a*^
UAs/Cr	–2.59 (–4.33, –0.86) *p* = 0.004	–2.42 (–4.15, –0.69) *p* = 0.006	–2.99 (–5.25, –0.74) *p* = 0.009	–1.38 (–3.08, 0.33) *p* = 0.11	–2.25 (–4.09, –0.40) *p* = 0.017
BMn	–6.54 (–11.42, –1.66) *p* = 0.009	–4.67 (–9.53, 0.19) *p* = 0.06	–8.91 (–15.24, –2.57) *p* = 0.006	–6.25 (–11.04, –1.46) *p* = 0.011	–2.95 (–8.14, 2.23) *p* = 0.26
*R*^2^	5.89%	4.25%	5.50%	3.49%	2.65%
Adjusted^*b*^
UAs/Cr	–1.81 (–3.30, –0.32) *p* = 0.018	–1.73 (–3.30, –0.16) *p* = 0.031	–2.03 (–4.03, –0.03) *p* = 0.047	–1.07 (–2.68, 0.54) *p* = 0.193	–1.40 (–3.03, 0.24) *p* = 0.095
BMn	–3.17 (–7.43, 1.10) *p* = 0.15	–1.66 (–6.15, 2.82) *p* = 0.47	–6.21 (–11.93, –0.49) *p* = 0.034	–3.55 (–8.15, 1.04) *p* = 0.13	0.03 (–4.65, 4.72) *p* = 0.99
*R*^2^	34.66%	25.89%	30.05%	19.32%	27.69%
^***a***^Model includes log_e_-UAs/Cr and log_e_-BMn only. ^***b***^Additionally adjusted for maternal IQ score and maternal age; HOME score; child’s school grade, head circumference, and plasma ferritin (categorized as low, high, or not available).					

For a difference in UAs/Cr between 161.24 (the baseline mean UAs/Cr in the low-WAs group) and 331.28 (the mean of UAs/Cr in the high-WAs group at baseline), Full Scale IQ, in Verbal Comprehension and Working Memory Indices were lower by 1.30, 1.25 and 1.46 points, respectively,

### Change in As and Mn Biomarkers and Changes in Intelligence

Final analyses examined associations between changes in As biomarkers (UAs/Cr and BAs) and follow-up IQ scores adjusting for baseline IQ and school grade. Adjusting for baseline Working Memory Index scores and for school grade at baseline, changes in UAs/Cr from baseline to followup were significantly related to changes in Working Memory Scores at follow-up (Table 4). For every decrease of 100 μg UAs/gCr, Working Memory scores increased by 0.91 points [SE = 0.39; 95% confidence interval (CI): 0.15, 1.67]. With adjustment for baseline intelligence and grade level, changes in UAs/Cr were not significantly related to changes in other areas of intellectual functioning. In parallel analyses using changes in blood biomarkers, neither BAs nor BMn was significantly associated with changes in intelligence test scores, although decreases in BAs remained modestly associated with increases in Working Memory (*p* = 0.06), even after adjustment for baseline Working Memory, baseline school grade, and changes in BMn (*p* = 0.07), with a 0.35-point increase (SE = 0.20) in Working Memory for 1-μg/L decrease in BAs (95% CI for points lost = 0.03, 0.74).

**Table 4:** Associations (coefficient and 95% CI) between a 100-μg/g increase in within-person change in UAs/Cr (*n* = 292), or a 1-μg/L increase in within-person change in BAs or BMn (*n* = 276), and changes in standardized WISC-IV scores between baseline and follow-up.

Exposure	∆ Full Scale	∆ Verbal Comprehension	∆ Working Memory	∆ Perceptual Reasoning	∆ Processing Speed
∆ UAs/Cr	–0.26 (–0.66, 0.15) *p* = 0.21	–0.04 (–0.55, 0.47) *p* = 0.88	–0.91 (–1.67, –0.14) *p* = 0.02	–0.01 (–0.51, 0.49) *p* = 0.97	–0.10 (–0.59, 0.40) *p* = 0.70
*R*^2^	15.74%	22.62%	9.11%	31.75%	25.18%
∆ BAs	–0.15 (–0.35, 0.05) *p* = 0.14	–0.13 (–0.39, 0.13) *p* = 0.32	–0.35 (–0.74, 0.03) *p* = 0.07	0.06 (–0.19, 0.32) *p* = 0.64	–0.17 (–0.42, 0.07) *p* = 0.17
*R*^2^	16.80%	22.95%	8.29%	30.94%	24.50%
All measures of change (∆) are calculated as follow-up level minus baseline level. Separate models were used to estimate associations with each exposure. All models are adjusted for school grade at baseline and for the IQ/Index score at baseline that corresponds to the outcome being assessed.					

## Discussion

After the installation of new deep wells, both UAs and UAs/Cr decreased significantly for the entire sample (decreases of approximately 20 μg/L and 115 μg/g), with decreases more marked in the high-As group, as expected. At follow-up, for both the high-As subsample and the full sample, the shorter the distance to a low-As well, the greater the decrement in children’s UAs/Cr; this association held for the distance to a well installed in the course of this investigation and for the distance to any deep, low-As well. Over the approximately 2 years between baseline and follow-up assessments, change in UAs/Cr (adjusting for baseline Working Memory Index scores and for school grade at baseline) was significantly related to change in Working Memory scores. Changes in other components of intelligence, however, were unrelated to changes in exposure.

The observed reductions in BAs and BMn indicate that the introduction of safe wells leads to reduced arsenic and manganese exposure: earlier, we reported that deep wells in our study region have low concentrations of both elements (van Geen et al. 2007). It is possible that the decline in BMn may be due in small part to the physiological decline in Mn bioavailablity with age (reviewed by Horning et al. 2015). Because the children’s mothers are the ones who bring water into the home, we speculate that all family members benefitted from the intervention. The reductions in exposure are likely not entirely attributable to our program of well installation and educational interventions, because other mitigation activities—including the installation of safe wells by the Bangladesh government—were ongoing during the course of the study. In another region of Bangladesh, we have previously shown that community participation in arsenic mitigation (well testing and education) can be effective in reducing exposure (George et al. 2012). Collectively, one cannot help but be heartened by the collective findings, though much wider efforts are still sorely needed, especially since the proportion of untested wells in the country continues to grow (van Geen et al. 2014).

The observed reductions in As and Mn exposure did not, for the most part, translate into improvements in child IQ. Studies of other health outcomes show similar patterns. For example, a carefully executed population-based case–control study in northern Chile found that elevated risks for lung and bladder cancer persisted 40 years after the cessation of As exposure (Steinmaus et al. 2013). In Bangladesh, short-term (4 years) reductions in water As exposure did not reduce overall mortality in the HEALS study (Argos et al. 2010). Impaired lung function also seems to persist long after the cessation of arsenic exposure early in life (Dauphiné et al. 2011). In the present study, the period between baseline and follow-up assessments was, on average, 2.79 years, with new wells installed across the first year of follow-up. It may be that a longer period of reduced As exposure is necessary to reverse decrements in IQ scores.

Other non-psychosocial interventions aimed at improving child IQ have reported mixed results. Perhaps the largest intervention to address an environmental exposure was the randomized, placebo-controlled trial of succimer (a lead-chelating agent) in 780 children with blood lead levels of 20–44 μg/dL (Rogan et al. 2001). Treatment with succimer successfully lowered blood lead levels, but no improvement in IQ test scores was observed. In contrast, the correction of nutritional deficiencies has had some success. For example, supplementation of iron in iron-deficient children has been observed to reverse developmental delays (Idjradlnata and Pollitt 1993; Wasserman et al. 1994).

Children whose exposure to WAs decreased over the 2 years of follow-up experienced reductions in biomarkers of exposure, and also showed significant gains in Working Memory scores. Of the 150 children in the high-WAs group, 132 experienced a decrease in UAs/Cr over the 2 years of follow-up (mean decrease, 202.90 μg UAs/gCr). For this subset, Working Memory scores were significantly improved by an (unadjusted) average of 2.23 points, [*t*_(131)_ = 2.19, *p* < 0.05; 95% CI: 0.24, 4.21].

In earlier work in Bangladesh, with children of similar ages, we reported negative associations between As measured in blood and WISC-IV Working Memory that remained marginally significant (*p* < 0.09) with adjustment for other contributors (Wasserman et al. 2011). In our previous cross-sectional study (Wasserman et al. 2014), after adjusting for other covariates, we found that average WISC-IV Working Memory scores were almost 5 points lower (–4.88; 95% CI: –9.30, –0.46) in children with WAs ≥ 5 μg/L compared with those with WAs < 5 μg/L.

In cognitive models, working memory is thought to provide “temporary storage and manipulation of the information necessary for . . . complex cognitive tasks” (Baddeley 1992), allowing individuals to retain certain task-related information while focusing on other aspects of the task. Working memory is key to many academic (e.g., adding long columns of numbers) and practical skills, such as retaining a phone number long enough to dial it. Working memory increases markedly across childhood, reaching levels comparable with those in adults by about age 15 years (Sander et al. 2012), reflecting growth in certain anatomical brain regions (such as frontal lobes) or in the functional connectivity of various circuitry. Strong associations have been noted between children’s working memory capacity and academic ability, literacy, and mathematics attainment.

It may be that working memory during childhood is particularly sensitive to environmental insults. On the other hand, there is evidence of impact of arsenic exposure on more generic aspects of memory in other developmental periods (and in other species). For example, among older adults (O’Bryant et al. 2011), relatively low WAs levels (mean WAs, 6.3 μg/L) were negatively associated with a wide range of cognitive skills, including processing speed, executive function, and memory. Animal studies have shown a dose-dependent accumulation of As in many parts of the brain (Sánchez-Peña et al. 2012; Wang et al. 2009) that play important roles in human cognition and memory. In As-exposed rodents, morphological and neurochemical changes have been noted in the hippocampus, along with learning and memory deficits (Luo et al. 2009; Martinez-Finley et al. 2009). Finally, results showed that use of standardized measures of intelligence identified a very similar set of baseline contributors to those reported in our earlier work with raw WISC-IV scores (Wasserman et al. 2011). Using standardized scores allows for comparisons with other global populations, and also allows specification of the degree of decrement attributable to changes in exposure.

### Limitations

We identified three limitations in this work. First, the follow-up period after the installation of a new well was short, so it may be that availability of low-As water for a longer period might demonstrate stronger impact on neurodevelopmental outcomes. Next, as the pace of arsenic mitigation activities in our study region has increased, we cannot attribute reductions in biomarkers solely to our installation of deep wells. Finally, we were unable to reliably define which well each child used at each point across follow-up. On the other hand, decreases in biomarkers of arsenic exposure at follow-up, and the greater decrease among those in the high WAs subgroup, indicate that children did switch to the newly installed and safer wells.

## Conclusions

The installation of low-As wells, coupled with an education initiative, can effectively reduce exposure to harmful levels of As and Mn in drinking water in Bangladesh. Over the relatively short term of this study (2 years), this exposure reduction was not associated with significant improvements in overall IQ scores, except for modest improvements in Working Memory; the possible impacts over longer periods of time remain to be seen. Clearly, the global health objective should be to prevent such exposures in the first place, so as to reduce the burden of a constellation of As-related diseases that are still ongoing in many parts of the world.
